# Pathological Feature and Immunoprofile of Cystitis Glandularis Accompanied with Upper Urinary Tract Obstruction

**DOI:** 10.1155/2014/872170

**Published:** 2014-05-29

**Authors:** Aihua Li, Jun Zhou, Honghai Lu, Xiaoming Zuo, Sikuan Liu, Feng Zhang, Weiwu Li, Wei Fang, Binghui Zhang

**Affiliations:** ^1^Department of Urology, Yangpu Hospital, School of Medicine, Tongji University, Shanghai 200090, China; ^2^Department of Urology, Huadong Hospital Affiliated with Fudan University, Shanghai 200040, China

## Abstract

*Objective*. To explore the pathological feature and immunoprofile of immunoprofile accompanied with upper urinary tract obstruction and the immunoprofile in various types of glandular cystitis. *Methods*. Pathological sections from 31 cases of cystitis glandularis with upper urinary tract obstruction and 34 cases of cystitis glandularis without upper urinary tract obstruction were observed as pathological feature on microscopy. Meanwhile, an immunohistochemical analysis was employed to determine the expression of p53, Ki67, p21, MMP-9, MUC1, MUC2, and COX-2. *Results*. In the two groups, main pathological type was transitional epithelial, followed by intestinal epithelial; other types were a few, and the difference between the two groups was not significant. All immunohistochemical expressions of p53, Ki67, p21, MMP-9, MUC1, MUC2, and COX-2 were positive in varying degrees, and there was no significant difference between the groups. Transitional epithelial type was compared with mixed type; the difference of COX-2 was significant, *P* < 0.05. The differences of immunohistochemical expression among other different pathologic types were not significant. *Conclusions*. It is suggested that glandular cystitis accompanied with upper urinary tract obstruction shares the same pathological feature and immunoprofile as that without upper urinary tract obstruction. No significant differences of immunohistochemical expression in tissue are in cystitis glandularis with different pathological types.

## 1. Introduction

Cystitis glandularis is a change that occurs in the tissue that lines the bladder. This particular type of tissue change is benign, but it can be a symptom of another health problem that needs to be treated. In addition, some cases of this condition precede the development of cancerous growths. For this reason, this finding can be a cause for concern. Our previous study found that cystitis glandularis could be induced by upper urinary tract obstruction and cystitis glandularis accompanied with upper urinary tract obstruction is associated with upper urinary tract obstruction [1, 2]. Several studies suggested that the expression of p53, Ki67, p21, MMP-9, MUC1, MUC2, and COX-2 in tissue will be increased in the process that cystitis glandularis evolves to bladder cancer [3–9]. In the study, pathological features of cystitis glandularis accompanied with upper urinary tract obstruction were observed; meanwhile, the immunoprofile was observed by measuring the expression of p53, Ki67, p21, MMP-9, MUC1, MUC2, and COX-2 in cystitis glandularis tissue.

## 2. Materials and Methods

### 2.1. Patients

The pathological sections of 65 cases diagnosed as cystitis glandularis from February 2006 to August 2011 in our hospital were evaluated. The biopsy specimens were obtained by cystoscopy. Patient age was from 28 to 86 years, with a mean age of 62.97 years. Among them, 13 cases were male and other 42 cases were female. Thirty- one cases were accompanied with upper urinary tract obstruction and 34 cases were accompanied without upper urinary tract obstruction. Pathological types were observed under light microscope; meanwhile, an immunohistochemical analysis was employed to determine the expression of p53, Ki67, p21, MMP-9, MUC1, MUC2, and COX-2.

### 2.2. Pathological Type

Tissue blocks were conventionally resected from the inflammatory lesions on bladder for preparation as microscopic specimens. The tissue blocks were fixed in 10% neutral formalin and conventionally dehydrated. Blocks were embedded in paraffin, cut at 3-4 *μ*m thickness, and then stained with hematoxylin and eosin (H-E) for examination under a light microscope and the pathological type was divided by transitional epithelial, intestinal epithelial, prostatic epithelial, and mixed type.

### 2.3. Immunohistochemical Technique

Primary antibodies of p53 (clone:D0-7), Ki67 (clone:sp6), p21 (clone:DSC-60.2), MMP-9 (clone:2C3), MUC1 (clone:ZM35), MUC2 (clone:Ccp58), and COX-2 (clone:cx-294) were purchased from Gene Tech (Shanghai) Company Limited, which were used without dilution. The secondary antibodies, ChemMate Envision Detection Kit of goat against rabbit and mouse antibody, were obtained from DaKo.

Standard two-step Envision method of immunohistochemical staining was used to assess the expressions of p53, Ki67, p21, MMP-9, MUC1, MUC2, and COX-2. Blocks were cut conventionally at 3-4 *μ*m thickness, dried and deparaffinized in xylene for 3 times, every time 15 minutes, and then rehydrated in graded ethanol. Endogenous peroxidase was blocked with 3% hydrogen peroxide; subsequently the sections were washed by distilled water and heated in a citrate buffer (pH 6.0) using a steam cooker at 95°C for 30 min. After being cooled, the sections were mounted by fetal bovine serum for 60 minutes, and then primary antibody was added and kept in wet box at 4°C for 24 hours. Sections were washed by Tris Buffered Saline for three times, every time for 5 minutes [10].

The secondary antibody was added and incubated for 30 minutes and then washed by Tris Buffered Saline for three times, every time for 5 minutes. Diaminobenzidine substrate solution was used as a chromogen and chromogenic time was controlled under a microscope. Then the slides were counterstained with hematoxylin, washed and dehydrated in ethanol, cleared in xylene, and finally mounted by gum. For the negative control, the buffer replaced the primary antibody. Positive staining patterns were compared with the known positive plates and other procedures follow the same step.

### 2.4. Interpretation of Immunohistochemical Staining Results

**Figure 1:**
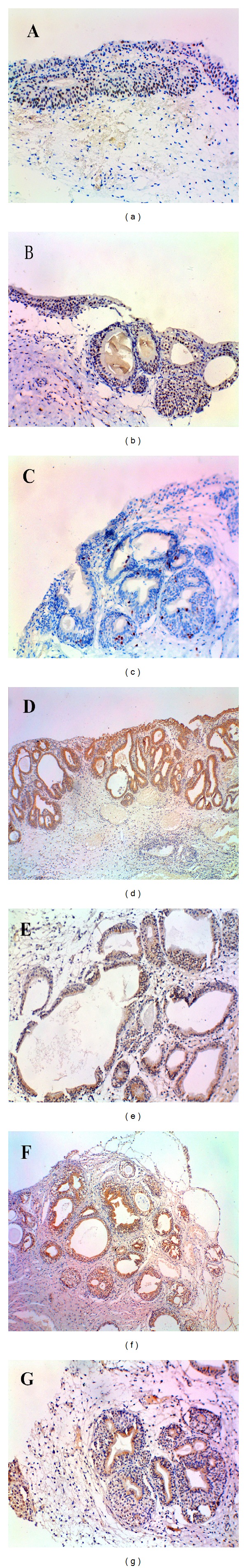
Immunohistochemical analysis of cystitis glandularis. (a) demonstrates positive nuclear staining for p53; (b) demonstrates positive nuclear staining for p21; (c) demonstrates positive nuclear staining for Ki-67; (d) demonstrates positive cytoplasmic staining for MMP-9; (e) demonstrates positive cytoplasmic staining for MUC2; (f) demonstrates positive cytoplasmic staining for COX-2; (g) demonstrates positive cytoplasmic staining or luminal membrane staining for MUC1.

Nuclear staining of p53, p21, and Ki-67, cytoplasmic staining of MMP-9, MUC2, and COX-2, and cytoplasmic staining or luminal membrane staining of MUC1 were considered positive (Figure 1).

We used a semiquantitative analysis system to determine immunostaining score. In brief, the scoring system was established according to the percentage and intensity of positive cells. For percentage, 0~4 scores represent <5%, 5~25%, 26~50%, 51~75%, and >75% of labeled cells, respectively. For the intensity, 0~3 score indicates weak, middle, and strong staining, respectively. Multiplication of both scores decided the final quotation, ranging 0~12, negative (0), weak positive (1–4, +), positive (5–8, ++), and strong positive (9–12, +++) [10].

A double-blind analysis was performed by two independent pathologists. Each sample was scored twice. All the slides were analyzed without knowledge of clinical data.

### 2.5. Ethical Consent

The study was approved by the Medical Ethics Committee of Yangou Hospital, School of Medicine, Tongji University.

### 2.6. Statistical Analysis

The differences between 2 analyzed data were compared using the *χ* ^2^ test and Student's *t*-test. Difference was considered significant at a *P* value < 0.05.

## 3. Results

In 32 cases with upper urinary tract obstruction, 26 cases (81.25%) were accompanied with ureteral calculi, 6 cases (18.75%) were accompanied with ureteral stenosis, 4 cases (12.50%) were accompanied with ureteropelvic junction obstruction, 2 cases (6.25%) were accompanied with ureteral deformity, 2 cases (6.25%) were accompanied with hematuria, and 6 cases (18.75%) were accompanied with positive urine culture. In 34 cases with upper urinary tract obstruction, 7 cases (20.59%) were accompanied with benign prostate hyperplasia, 3 cases (8.82%) were accompanied with urethra stenosis, 1 case (2.94%) was accompanied with prostate cancer, 1 case (2.94%) was accompanied with bladder stone, 1 case (2.94%) was accompanied with bladder tumor, 15 cases (44.11%) were accompanied with hematuria, and 8 cases (32.53%) were accompanied with positive urine culture.

On microscopy, main pathological type of cystitis glandularis was transitional epithelial, followed by intestinal epithelial; prostatic epithelial and mixed types were a few. In addition, squamous metaplasia, Von Brunn's nests, and inflammatory cell infiltration would appear among them in some pattern. Patient age was 57.25 ± 9.92 years in group with upper urinary tract obstruction and 68.35 ± 12.86 years in group without upper urinary tract obstruction, *P* > 0.05.

## 4. Discussion

Cystitis glandularis is considered benign but with uncertain malignant potential. Several authors have described the progression to bladder adenocarcinoma during long-term follow-up, especially; intestinal metaplasia is most commonly considered as a risk factor and a putative precursor of adenocarcinoma, although others have disputed this finding [1, 2, 11]. Our previous study found that cystitis glandularis cloud be induced by upper urinary tract obstruction [1, 2]. In the study, cystitis glandularis was histologically divided into four types, transitional epithelial, intestinal epithelial, prostatic epithelial, and mixed type. Transitional epithelial type was the main pathological type; intestinal epithelial type was the second. The difference of pathological type between cystitis glandularis with and without upper urinary tract obstruction was not significant (Table 1).

Ki67 antigen is a nonhistone protein, presented during the cell cycle phases G1, S, G2, and M, being an established indicator of tumor growth and aggressiveness. The well-known tumor suppressor p53, which functions primarily as a transcription factor, plays a vital role in protecting cells from a variety of cellular stresses, including DNA damage and oncogene activation. In previous studies, increased Ki67 and p53 expression was proven to be related to tumor grade, stage, recurrence, progression, and survival of bladder cancer [12–14]. p53 showed good correlation with histologic progression and histologic grade and Ki67 was strongly associated with recurrence and histologic grade in bladder tumor [15]. p21 is a potent cyclin-dependent kinase inhibitor and functions as a regulator of cell cycle progression at G1 and S phases. The expression of this gene is tightly controlled by the tumor suppressor protein p53, through which this protein mediates the p53-dependent cell cycle G1 phase arrest in response to a variety of stress stimuli [16, 17]. Previously study demonstrates that positive expression of Ki67, p53, and p21, especially when simultaneously assessed, has independent prognostic value on bladder tumor. Other studies suggested that the expression of Ki67 in cystitis glandularis was similar to low grade of transitional cell carcinoma of bladder, lower than high grade of transitional cell carcinoma of bladder, and higher than normal bladder. Cystitis glandularis is a benign disease with obvious possibility of malignant change [3]. P53 gene may play an important role in the canceration of cystitis glandularis and the p53 and Ki-67 expression levels were reduced significantly after treatment of cystitis glandularis [4, 5]. P21 protein is a good index of glandular bladder transitional inflammatory changes to the pain and provides information for early diagnosis of bladder cancer [6].

MMP-9 (matrix metallopeptidase 9) is an enzyme that in humans is encoded by the MMP-9 gene. MMP-9 can be involved in the development of several human malignancies, as degradation of collagen IV in basement membrane and extracellular matrix facilitates tumor progression, including invasion, metastasis, growth, and angiogenesis [18]. Mucins constitute a large family of glycoproteins expressed by many epithelial cells and their malignant counterparts. MUC1 is a transmembrane glycoprotein that is known to have a role in lumen formation and has an inhibitory role in the cell-to-stroma interaction. It can be identified in a wide range of secretory epithelia and their neoplastic equivalents. The gel-forming mucin, MUC2, is found in a cluster on chromosome 11p15.5. The MUC2 gene codes for a typical secretory mucin that predominates in goblet cells of the small and large intestine [11]. Ma et al. [7] found that the positive expression rate of MMP-9 in cystitis glandularis was significantly higher than normal tissues and the positive expression rate in recurrent cystitis glandularis was significantly higher than nonrecurrent cystitis glandularis. Xiao et al. [8] showed that strong expression of MUC1 gene was observed only in cystitis glandularis and adenocarcinoma. The straining patterns were significantly associated with cell hyperplasia and tumor cell differentiation. The highly expressed MUC1 could play an important role in tumor infiltration and metastasis. In the study, all immunohistochemical expressions of p53, Ki67, p21, MMP-9, MUC1, and MUC2 from different pathological types were positive in different degree in the two groups (Table 2). But there were no significant differences between the two groups and between different pathological types of cystitis glandularis.

Cyclooxygenase-2 (COX-2) is regarded as induced inflammatory mediator involved in the development of tumors. It is an inducible enzyme (also called prostaglandin syntheses) responsible for conversion of arachidonic acid to prostaglandins and other inflammatory mediators. It is not detectable in most normal tissues; however, it is induced at sites of inflammation by cytokines, growth factors, and tumor promoters. Also, prominent COX-2 expression has been described in bladder cancers including transitional cell and squamous cell carcinomas and this expression correlates with tumor grade and invasion [18–20]. Jiang et al. [9] hinted that the COX-2 expression in cystitis glandularis was higher than normal tissue and the rate and intensity in the recurrent cases were significantly higher, which indicated that the clinical detection of COX-2 was of a guiding significance to judge the prognosis of cystitis glandularis. In the study, the expression of COX-2 was positive in the two groups but the difference between the two groups was not significant. And transitional epithelial was compared with mixed type and the difference was significant. However, there was no significant difference between other different pathological types of cystitis glandular.

## 5. Conclusion

It is suggested that glandular cystitis accompanied with upper urinary tract obstruction shares the same pathological feature and immunoprofile as that without upper urinary tract obstruction. Meanwhile, immunohistochemical expressions of P53, Ki67, p21, MMP-9, MUC1, MUC2, and COX-2 in cystitis glandularis with or without upper urinary tract obstruction have changed. The differences of immunohistochemical expressions in different types of cystitis glandularis and accompanied with or without upper urinary tract obstruction were not significant (Table 3). It is hard to anticipate the malignant tendency of glandular cystitis by the determination of immunohistochemical expression in tissue. Therefore, to the cystitis glandularis accompanied with upper urinary tract obstruction, the most important thing is to identify and remove the causes of upper urinary tract obstruction. Cystitis glandularis should be actively treated when the lesions are more serious; in other cases, long-term close follow-up should always be done.

## Figures and Tables

**Table 1:** Pathological type in cystitis glandularis with and without upper urinary tract obstruction.

	With upper urinary tract obstruction (*n* = 31)	Without upper urinary tract obstruction (*n* = 34)
Transitional epithelial	17 (54.84%)	21 (61.76%)
Intestinal epithelial	9 (29.03%)	7 (20.59%)
Prostatic epithelial	2 (6.45%)	0
Mixed	3 (9.68%)	6 (17.65%)

Transitional epithelial type was the main pathological type of cystitis glandularis; intestinal epithelial type was the second; prostatic epithelial and mixed types were fewer. The differences were not significant between the two groups.

**Table 2:** Immunohistochemical expression of cystitis glandularis in different pathological types.

	Transitional epithelial	Intestinal epithelial	Prostatic epithelial	Mixed
*N*	38	16	2	9
P53	19 (50.00%)	8 (50.00%)	1 (50.00%)	3 (33.33%)
Ki67	15 (39.47%)	5 (31.25%)	0	3 (33.33%)
P21	28 (73.68%)	9 (56.25%)	1 (50.00%)	6 (66.67%)
MMP-9	21 (55.26%)	8 (50.00%)	1 (50.00%)	3 (33.33%)
MUC1	17 (44.74%)	8 (50.00%)	1 (50.00%)	2 (22.22%)
MUC2	6 (15.79%)	6 (37.50%)	0	0
COX-2	26 (68.42%)	10 (62.50%)	1 (50.00%)	2 (22.22%)

All immunohistochemical expressions of p53, Ki67, p21, MMP-9, MUC1, MUC2, and COX-2 from different pathological types in the two groups were positive in different degree. Transitional epithelial type was compared with mixed type; the difference in expression of COX-2 was significant, *P* < 0.05. However, there was no significant difference between other different pathological types of cystitis glandular.

**Table 3:** Immunohistochemical expression of cystitis glandularis with and without upper urinary tract obstruction.

	With upper urinary tract obstruction (*n* = 31)				Without upper urinary tract obstruction (*n* = 34)			
	0	+	++	+++	0	+	++	+++
P53	14	12	5	0	20	11	2	1
Ki67	18	10	3	0	24	6	2	2
P21	11	11	7	2	10	15	9	0
MMP-9	16	9	5	1	16	7	10	1
MUC1	19	10	2	0	18	13	3	0
MUC2	26	4	1	0	27	6	0	1
COX-2	13	14	4	0	13	12	5	4

Compared between the two groups, the differences were not significant.
